# Towards precision management of *Mycoplasma genitalium*: a real-world cohort study identifying key predictors for treatment failure and the superiority of sequential therapy

**DOI:** 10.3389/fcimb.2026.1787520

**Published:** 2026-04-02

**Authors:** Xinmin Qiu, Baobing Chen, Jianping Chen, Xinzheng Li, Jiyun Tian

**Affiliations:** Department of Clinical Laboratory, Hangzhou Third People’s Hospital, Hangzhou, China

**Keywords:** *Chlamydia trachomatis* (CT) co-infection, *Mycoplasma genitalium* (MG), real-world evidence, risk stratification, sequential therapy, treatment failure

## Abstract

**Background:**

The management of *Mycoplasma genitalium* (MG) infection is challenged by rising macrolide resistance, leading to high failure rates with azithromycin. Evidence on the long-term effectiveness of alternative initial regimens, particularly sequential therapy, and easily obtainable predictors for poor outcomes remains scarce.

**Methods:**

We conducted a retrospective cohort study at a tertiary hospital in China (2018-2024). Sexually active adults with nucleic acid amplification test (NAAT)-confirmed MG infection and available treatment records were included. The primary outcomes were treatment failure (persistent infection at 8 weeks, ultimate failure) and recurrence. Multivariable logistic regression and Kaplan-Meier survival analyses were employed to assess the impact of initial antibiotic regimens (azithromycin, quinolones, doxycycline-quinolone sequential therapy), demographics, and co-infections.

**Results:**

Among 1, 192 MG-positive patients, 474 completed follow-up. After adjustment, doxycycline-quinolone sequential therapy was associated with significantly lower odds of ultimate treatment failure (adjusted odds ratio [aOR]=0.36, 95%CI:0.21-0.61) and recurrence (aOR=0.37, 95%CI:0.18-0.75) compared to azithromycin. Survival analysis confirmed a faster median time to clearance with sequential therapy (7 vs. 10 weeks, p=0.001) and a marked “long-tail” effect in the azithromycin group (mean clearance time: 94.2 vs. 28.0 weeks). Co-infection with *Chlamydia trachomatis* (CT) was the strongest independent predictor for all adverse outcomes (aOR for ultimate failure=3.21, 95%CI:1.88-5.48), followed by male sex.

**Conclusions:**

In this real-world cohort, doxycycline-quinolone sequential therapy demonstrated superior long-term effectiveness over azithromycin for MG infection. CT co-infection and male sex were key risk predictors. These findings advocate for a paradigm shift towards risk-stratified initial therapy, prioritizing sequential regimens for high-risk patients to improve cure rates while supporting antimicrobial stewardship.

## 1 Introduction

*Mycoplasma genitalium* (MG), a cell-wall-deficient, sexually transmitted pathogen, is increasingly recognized as a primary etiological agent of non-gonococcal urethritis (NGU) and cervicitis, following advances in molecular diagnostics (McGowin and Anderson-Smits, 2011; Horner and Martin, 2017). Diagnosing MG infection is notoriously difficult due to its slow growth and fastidious culture requirements, which are impractical for routine clinical use. Consequently, nucleic acid amplification tests (NAATs) have become the gold standard for diagnosis (Taylor-Robinson and Jensen, 2011; Jensen et al., 2022). Specifically, RNA-based simultaneous amplification and testing (RNA-SAT) targeting 16S rRNA offers high sensitivity and specificity (Liang et al., 2018). In addition, because RNA is generally less persistent than DNA after organism death, RNA-based assays may be less influenced by residual nucleic acids when interpreting post-treatment positivity (Cangelosi and Meschke, 2014).

Global epidemiological data indicate a wide-ranging prevalence of MG ranging from 1.3% to 15.9% across different populations, representing a substantial disease burden in China as well (Taylor-Robinson and Jensen, 2011; Baumann et al., 2018; Gnanadurai and Fifer, 2020). Beyond causing acute urethritis and cervicitis, MG infection is a key contributor to pelvic inflammatory disease (PID), tubal factor infertility, ectopic pregnancy, and adverse reproductive outcomes such as preterm birth and spontaneous abortion, posing a persistent and serious public health challenge (Gnanadurai and Fifer, 2020; Olson et al., 2021). The infection disproportionately affects sexually active individuals, with prevalence varying significantly by gender, age, and sexual behaviors. Notably, epidemiological studies reveal that MG infection frequently coexists with other sexually transmitted pathogens such as *Chlamydia trachomatis* (CT), *Ureaplasma urealyticum* (UU), and *Neisseria gonorrhoeae* (NG), with co-infection rates reaching 10–30% across populations (Begnis et al., 2021; Richardson et al., 2021; Zhang et al., 2021). This pattern of co-infection not only reflects a shared background of high-risk behavioral exposure but may also directly influence the colonization and clearance dynamics of MG through complex microbe-host interactions (Darville and Hiltke, 2010). Yet, much of the existing literature on MG co-infection has focused on describing its epidemiological patterns, rather than evaluating its prognostic implications for treatment outcomes (Che et al., 2022; Lillis et al., 2026). Consequently, the clinical value of co-infection status as an independent marker to guide therapy remains underexplored. We therefore hypothesize that specific co-infection patterns (such as those involving CT, NG, or UU) could serve as efficient and readily available clinical indicators for the early identification of patients at high risk for MG treatment failure and recurrence.

Current treatment guidelines strongly advocate resistance-guided therapy because of the alarming global rise in macrolide resistance, which has exceeded 50% in many settings in recent years and has rendered empirical azithromycin monotherapy increasingly ineffective (Machalek et al., 2020; Workowski et al., 2021; Jensen et al., 2022). However, implementing resistance testing remains challenging in many resource-limited settings, including China, necessitating optimized empirical strategies. Sequential therapy (e.g., doxycycline followed by moxifloxacin) has emerged as a potential solution to mitigate bacterial load and improve cure rates (Workowski et al., 2021; Jensen et al., 2022; Chinese Academy of Medical Sciences Hospital of Dermatology et al., 2024). Nevertheless, current decisions regarding these alternative regimens—particularly sequential therapy—still rely heavily on evidence of short-term microbiological clearance, often derived from studies with limited scale (Raccagni et al., 2023; Yuan et al., 2025; Ramchandani et al., 2025).

Utilizing extensive retrospective data from a large clinical cohort, this study aims to address two critical questions: (1) Do demographic factors and initial antibiotic regimens (azithromycin, quinolones, and sequential therapy) differ in their impact on short-term clearance and recurrence risk? (2) Are specific co-infection patterns independent predictors of adverse treatment outcomes? Through this analysis, we seek to provide evidence for more precise empirical treatment strategies in settings where resistance testing is unavailable.

## 2 Patients and methods

### 2.1 Patients

#### 2.1.1 Patients inclusion

This retrospective cohort study was conducted at Hangzhou Third People’s Hospital. Between January 2018 and December 2024, we systematically screened electronic medical records of patients from the Dermatology (Venereology), Urology, and Gynecology departments who had undergone RNA-SAT for MG. Concurrent testing for UU, CT, and NG was performed in the majority of cases based on clinical indication. Testing indications included symptoms suggestive of genitourinary tract infection—such as urethral or vaginal discharge, dysuria, pelvic discomfort, or genital pruritus—or routine screening following high-risk sexual behavior or contact with an infected partner. Eligible participants were sexually active adults aged 18–80 years. We excluded individuals with no sexual history, those who had used antibiotics within two weeks prior to testing, and pregnant or lactating women. From this screened population, patients with a positive MG test and available treatment records constituted the core analytic cohort for assessing treatment outcomes, while MG-negative patients formed a comparison group for baseline epidemiological description.

This retrospective study was reviewed and approved by the Institutional Review Board/Ethics Committee of Hangzhou Third People’s Hospital (Approval Number: 2026KA029). The requirement for informed consent was waived due to the retrospective nature of the study using anonymized data.

#### 2.1.2 Treatment regimens and follow-up

Initial antibiotic regimens were categorized based on the prescribed medications. (1) Doxycycline-quinolone sequential therapy: Oral doxycycline 100 mg twice daily for 7 days, followed by oral moxifloxacin or levofloxacin 400 mg once daily for 7 days. (2) Quinolone: Oral moxifloxacin or levofloxacin 400 mg once daily for 7 days. (3) Azithromycin: Oral azithromycin 1 g as a single dose on day 1, followed by 500 mg once daily on days 2–4. (4) Others: This category encompassed various non-standard regimens, including cephalosporins, clindamycin, and proprietary Chinese herbal preparations (e.g., Jinyinhua [Lonicera japonica] granule).

Following the completion of antimicrobial therapy, patients were advised to return for a test of cure (TOC) three weeks later. Due to the retrospective nature of the study, actual follow-up intervals varied based on patient adherence; however, the majority of TOCs were performed within a window of two to six weeks post-treatment.

#### 2.1.3 Data collection and outcome definitions

Demographic data, clinical variables (e.g., age, sex, co-infections, treatment regimens), and key clinical outcomes were systematically extracted from electronic medical and laboratory records. Persistent infection was defined as having exclusively MG-positive test results throughout the entire follow-up period with no documented clearance. Recurrence was defined as a positive RNA-SAT result occurring after a confirmed negative TOC. Due to the lack of molecular genotyping in routine clinical practice, this operational definition encompasses both biological relapse (persistence of the original strain) and potential reinfection (acquisition of a new strain). The duration of positivity was calculated in weeks from treatment initiation to the first documented MG-negative test for patients achieving clearance, or to the date of the last MG-positive test for those not cleared.

Because this was a retrospective study based on routine clinical documentation, certain variables were not systematically recorded, such as sexual orientation and specific sexual behaviors. Additionally, molecular testing for macrolide resistance-associated mutations (23S rRNA and *parC*) was not part of standard care during the study period; thus, resistance genotype data were unavailable.

loss to follow-up (LTFU) was defined as having no further clinical visit or laboratory records for more than 90 days after initial treatment. For efficacy assessments, a complete case analysis approach was utilized. Patients without valid TOC results or those LTFU were excluded from cure rate calculations. To assess potential selection bias, baseline characteristics were compared between patients included in the final analytic cohort and those lost to follow-up.

### 2.2 Methods

#### 2.2.1 Sample preparation

Specimen collection followed established clinical guidelines to ensure high diagnostic yield while maintaining patient acceptability, using specimen types previously validated for the RNA-SAT platform in our clinical setting (Zhong et al., 2015; Workowski et al., 2021; Jensen et al., 2022). For male patients, first-void urine (FVU; ≥2 hours urinary retention) was the primary specimen collected. For female patients, endocervical swabs were the standard procedure, with FVU serving as an alternative for those declining pelvic examinations. When swab specimens were collected, urethral (male) or endocervical (female) swabs were inserted 1–2 cm, rotated three times clockwise, retained for 15 seconds, and withdrawn. Swabs were immersed in 1 mL sterile saline and expressed against collection tube walls to release absorbed fluid. All specimens were immediately mixed 1:1 with RNA-stabilizing solution (kit-provided) for preservation.

#### 2.2.2 RNA detection of MG, UU, CT and NG

Clinical specimens (urine, urethral/cervical swabs) were tested using the RNA-SAT assay (Shanghai Rendu Biological Technology Co., Ltd.), which targets the 16S rRNA gene of MG, CT, NG and UU. This commercial assay utilizes an integrated system where all necessary reagents—including the RNA-stabilizing sample preservation solution, magnetic beads, lysis buffer, wash solutions, and amplification master mix—are provided by the manufacturer. In accordance with the manufacturer’s instructions, a total of 400 μL of preserved samples underwent nucleic acid extraction via magnetic bead purification, involving lysis at 60 °C and two subsequent wash steps using the kit-supplied buffers. This was followed by target-specific amplification, which consisted of 40 cycles at 42 °C, with real-time fluorescence detection conducted at the end of each cycle. Results were considered positive if the cycle threshold (Ct) was ≤35. Each run was validated with internal controls.

### 2.3 Statistical analyses

All analyses were performed using SPSS Statistics software for Windows, version 23.0 (IBM Corp, Armonk, NY, USA). A two-sided p-value of <0.05 was considered statistically significant. In descriptive and univariate analysis, categorical variables were presented as numbers and percentages, and compared using the Chi-square test or Fisher’s exact test, as appropriate. Continuous variables, which were non-normally distributed as assessed by the Shapiro-Wilk test, were presented as medians with interquartile ranges (IQR) and compared using the Mann-Whitney U test.

To identify independent factors associated with different treatment outcomes, three separate multivariable binary logistic regression models were constructed with the following dependent variables: 1) persistent infection at the 8-week follow-up (yes/no), 2) ultimate treatment failure (yes/no), and 3) recurrence among initially cleared patients (yes/no). For all models, independent variables included age (as a continuous variable), sex, baseline co-infection status (UU, CT, NG, each as a binary variable), and the initial antibiotic regimen (with azithromycin as the reference category). Results were reported as adjusted odds ratios (aOR) with 95% confidence intervals (CI). Time to MG clearance was analyzed using Kaplan-Meier survival curves, with comparisons between groups made by the log-rank test.

## 3 Results

### 3.1 Study population and baseline characteristics

Between 2018 and 2024, a total of 26, 672 individuals underwent syndromic testing for UU, CT, NG, and MG. The overall prevalence of MG infection was 4.5% (1, 192/26, 672). As shown in **Table 1**, MG-positive patients were significantly younger than MG-negative individuals (median age 31 vs. 34 years, p<0.001) and were more likely to be male (65.2% vs. 55.4%, p<0.001). Co-infection with other sexually transmitted pathogens was common among MG-positive patients, with significantly higher rates of UU (49.1% vs. 44.3%, p=0.001) and CT (22.4% vs. 10.4%, p<0.001) compared to the MG-negative group (**Table 1**).

**Table 1:** Baseline characteristics of the study population and the MG positive cohort by follow-up status.

Factor	MG-negative (n=25480)	MG-positive (n=1, 192)	p1	MG positive		
				Completed follow up (474)	Lost to follow-up(718)	p2
Age, years, median (IQR)	34(27-44)	31(25-38)	**<0.001**	31(26-39)	31(24-38)	**0.041**
Sex, n (%)						
Male	55.4%(14127/25480)	65.2%(777/1192)	**<0.001**	68.6%(325/474)	63.0%(452/718)	**0.047**
Female	44.6%(11353/25480)	34.8%(415/1192)		31.4%(149/474)	37.0%(266/718)	
Co-infection, n (%)						
*U. urealyticum*	44.3%(11195/25297)	49.1%(578/1177)	**0.001**	43.9%(205/467)	52.5%(373/710)	**0.004**
*C. trachomatis*	10.4%(2352/22664)	22.4%(248/1108)	**<0.001**	21.2%(94/444)	23.2%(154/664)	0.462
*N. gonorrhoeae*	7.6%(1553/20430)	8.7%(93/1063)	0.172	7.4%(32/433)	9.7%(61/630)	0.224

IQR, interquartile range; MG, *Mycoplasma genitalium*.

p1 refers to the comparison between MG negative and MG positive individuals; p2 refers to the comparison between MG positive patients who completed follow-up and those lost to follow-up.

Continuous variables were compared using the Mann–Whitney U test, and categorical variables were compared using the Chi-square test.

Percentages are calculated based on available data for each variable; denominators vary slightly due to missing test results.

Bold values indicate P<0.05, considered statistically significant.

**Figure 1:**
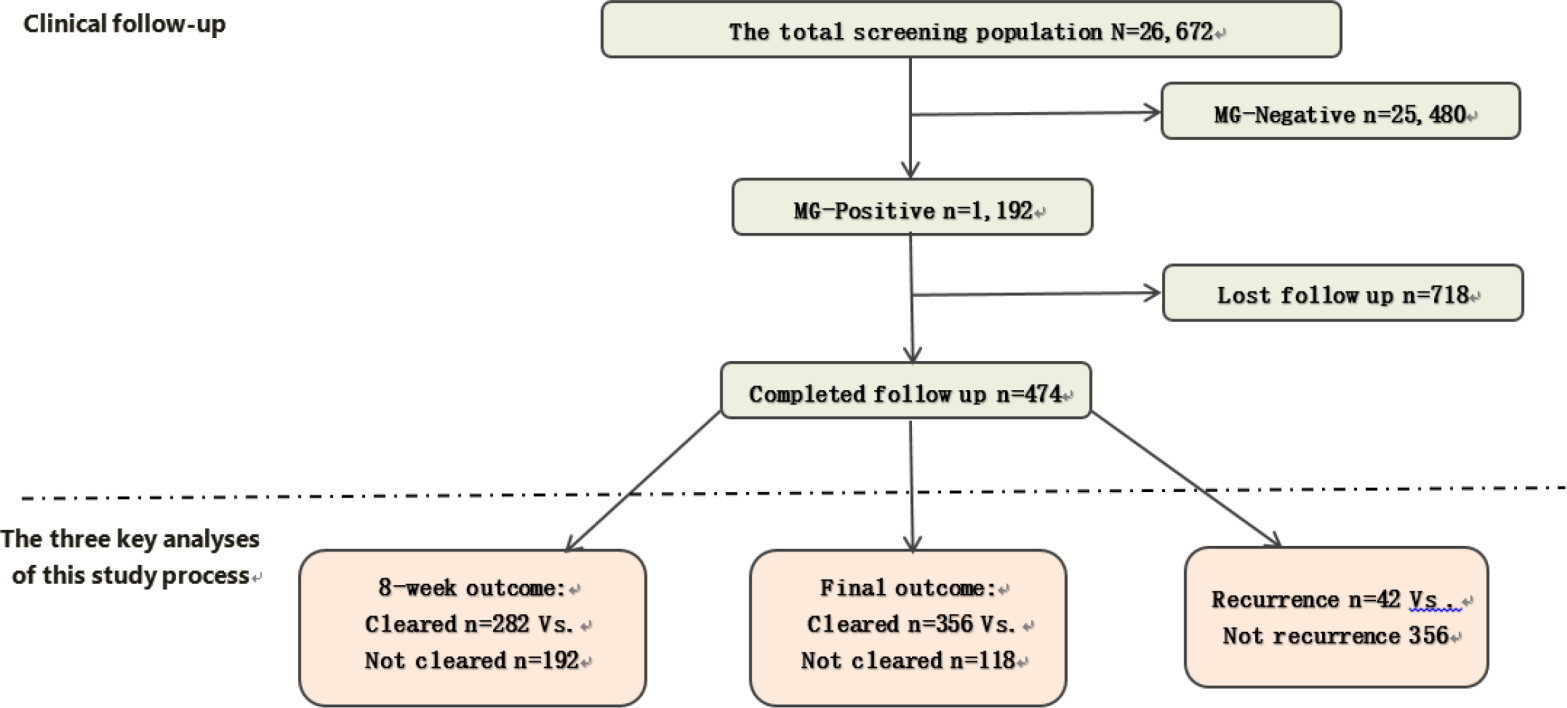
Study cohort flow diagram and outcome subgroup composition. **Figure 1** illustrates the derivation of the study population and the subgroups for primary outcome analyses. A total of 26, 672 individuals were screened, of whom 1, 192 tested positive for *M. genitalium* (MG). Among MG-positive patients, 474 completed follow-up and constituted the core analytic cohort. The three key analytic comparisons are shown: (1) early clearance at 8 weeks (n=282 cleared vs. n=192 not cleared); (2) ultimate treatment outcome (n=356 cleared vs. n=118 not cleared); and (3) recurrence among initially cleared patients (n=42 recurrence vs. n=432 without recurrence).

Among the 1, 192 MG-positive patients, 474 (39.8%) completed the follow-up protocol and constituted the primary cohort for treatment outcome analysis, while 718 (60.2%) were lost to follow-up (**Figure 1**). Those lost to follow-up were slightly younger (p = 0.041), more likely to be female (37.0% vs. 31.4%, p = 0.047) and had a higher proportion of UU co-infection (52.5% vs. 43.9%, p = 0.004). No significant differences were found for CT or NG co-infection between follow-up groups (**Table 1**).

### 3.2 Factors associated with early treatment response at 8 weeks

The baseline characteristics of the 474 patients with follow-up, stratified by their 8-week treatment outcome, are presented in **Table 2**. 282 (59.5%) achieved microbial clearance of MG at 8 weeks, while 192 (40.5%) did not. In univariate analysis, patients who failed to clear the infection by 8 weeks were more likely to be male (76.6% vs. 63.1%, p=0.002) and to have baseline co-infection with CT (28.3% vs. 16.2%, p=0.003). There were no significant differences in age, UU or NG co-infection status, or the distribution of initial antibiotic regimens between the two groups.

**Table 2:** Baseline characteristics and initial management of MG positive patients with follow-up, by treatment outcome at 8 weeks.

Factor	Cleared (n=282)	Not cleared (n=192)	P value
Age, years, median (IQR)	32(26-39)	30(25-38)	0.115
Sex, n (%)			
Male	63.1%(178/282)	76.6%(147/192)	9.576
Female	36.9%(104/282)	23.4%(45/192)	**0.002**
Co-infection, n (%)			
*U. urealyticum*	45.3%(125/276)	41.2%(80/191)	0.507
*C. trachomatis*	16.2%(42/260)	28.3%(52/184)	**0.003**
*N. gonorrhoeae*	7.8%(20/256)	6.8%(12/177)	0.714
Initial Antibiotic, n (%)			
Azithromycin	56.5%(52/92)	43.5%(40/92)	0.892
Doxycycline-quinolone	60.4%(204/338)	39.6%(134/338)	
Quinolone	64.3%(17/30)	35.7%(13/30)	
others	59.5%(9/14)	40.5%(5/14)	

Abbreviations, statistical methods, and handling of missing data are as described in the footnote to **Table 1**.

### 3.3 Multivariable predictors of early treatment failure

Multivariable logistic regression was performed to identify independent predictors of failure to achieve 8-week MG clearance (**Table 3**). After adjusting for age, sex, co-infections, and initial antibiotic regimen, two factors remained significantly associated with an increased odds of early treatment failure: male sex (adjusted odds ratio [aOR] = 2.25, 95% CI: 1.34–3.78, p=0.002) and co-infection with CT (aOR = 2.34, 95% CI: 1.41–3.86, p=0.001). Increasing age was associated with a slight but significant reduction in the odds of persistent infection (aOR = 0.98 per year, 95% CI: 0.96–1.00, p=0.033). Notably, neither co-infection with UU or NG, nor the choice of initial antibiotic regimen (doxycycline-quinolone sequential therapy, quinolone, or other agents versus azithromycin) showed a statistically significant association with the 8-week outcome in the adjusted model.

**Table 3:** Multivariable logistic regression analysis of factors associated with MG clearance within 8 weeks.

Factor	Adjusted odds ratio (aOR)	95% confidence interval	P value
Age (per year increase)	0.98	0.96-0.99	**0.033**
Sex (Male vs. Female)	2.25	1.34-3.78	**0.002**
*U. urealyticum* co-infection (Yes vs. No)	0.93	0.59-1.45	0.738
*C. trachomatis* co-infection (Yes vs. No)	2.34	1.41-3.86	**0.001**
*N. gonorrhoeae* co-infection (Yes vs. No)	0.83	0.38-1.85	0.654
Initial antibiotic regimen (Ref: Azithromycin)			
Doxycycline-quinolone	1.01	0.61-1.68	0.966
Quinolone	1.14	0.46-2.78	0.779
others	0.98	0.27-3.51	0.97

The model included age, sex, baseline co-infection status, and initial antibiotic regimen. Azithromycin was used as the reference category for antibiotic regimen comparisons. Age was entered as a continuous variable per year increase. Data on sexual orientation and macrolide resistance-associated mutations were unavailable and thus not included in the model. Adjusted odds ratios (aOR) > 1 indicate a higher likelihood of failure to achieve clearance, whereas aOR < 1 indicate a lower likelihood.

Bold values indicate P<0.05, considered statistically significant.

### 3.4 Multivariable predictors of ultimate treatment failure

A multivariable logistic regression model was constructed to identify factors independently associated with ultimate treatment failure (**Table 4**). In the multivariable model, co-infection with CT emerged as the strongest risk factor for ultimate treatment failure, increasing the odds by more than three-fold (aOR = 3.21, 95% CI: 1.88–5.48, p<0.001). Male sex remained a significant, albeit weaker, risk factor (aOR = 1.94, 95% CI: 1.06–3.54, p=0.032). Most notably, initial therapy with doxycycline-quinolone sequential therapy was a powerful protective factor. Compared to patients who started treatment with azithromycin, those prescribed doxycycline-quinolone sequential therapy had a 64% lower odds of ultimately failing treatment (aOR = 0.36, 95% CI: 0.21–0.61, p<0.001). Neither age nor co-infection with UU or NG showed a significant independent association with the ultimate outcome. The effects of quinolone and other regimens were not statistically different from that of azithromycin. The observed difference in ultimate failure rates may be explained by the dynamics of clearance. We therefore analyzed the time to clearance among treated patients.

**Table 4:** Multivariable analysis of factors associated with ultimate treatment failure of MG infection.

Factor	Adjusted odds ratio (aOR)	95% confidence interval	P value
Age (per year increase)	0.98	0.96-1.01	0.149
Sex (Male vs. Female)	1.94	1.06-3.54	**0.032**
*U. urealyticum* co-infection (Yes vs. No)	0.72	0.43-1.21	0.217
*C. trachomatis* co-infection (Yes vs. No)	3.21	1.88-5.48	**<0.001**
*N. gonorrhoeae* co-infection (Yes vs. No)	0.70	0.27-1.83	0.469
Initial antibiotic regimen (Ref: Azithromycin)			
Doxycycline-quinolone	0.36	0.21-0.61	**<0.001**
Quinolone	0.44	0.16-1.18	0.103
others	0.43	0.100-1.82	0.249

Outcome definition (ultimate treatment failure) differs from that in **Table 3**, but variable definitions, model construction, and interpretation of adjusted odds ratios otherwise follow the footnote to **Table 3**.

Bold values indicate P<0.05, considered statistically significant.

### 3.5 Time to clearance of MG infection

**Figure 2:**
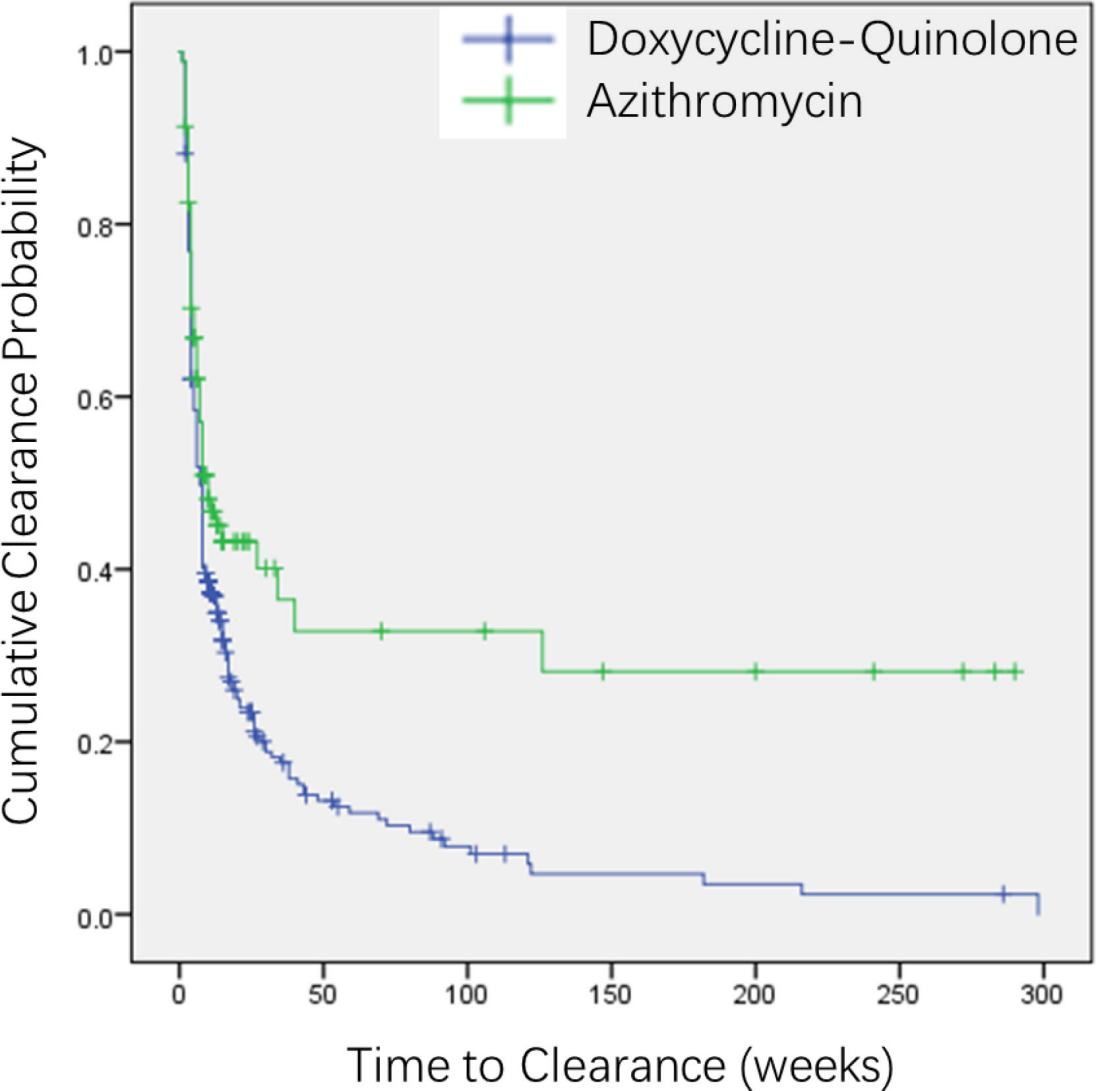
Kaplan–Meier curves comparing time to MG clearance between patients receiving initial doxycycline-quinolone sequential therapy (blue line) versus azithromycin monotherapy (green line). The median time to clearance was 7 weeks (95% CI: 6.1–7.9) for doxycycline-quinolone and 10 weeks (95% CI: 3.9–16.1) for azithromycin (Log-rank test, p = 0.001). The mean time to clearance was 28.0 weeks (95% CI: 19.5–36.4) and 94.2 weeks (95% CI: 58.5–129.8), respectively, indicating a pronounced “long-tail” of delayed clearance in the azithromycin group.

To further compare the long-term clearance dynamics between the two most common initial regimens, we conducted a Kaplan-Meier survival analysis focusing on doxycycline-quinolone sequential therapy (n=338) versus azithromycin (n=92), excluding smaller treatment groups to reduce noise. The median time to documented MG clearance was 7 weeks (95% CI: 6.1–7.9 weeks) for the doxycycline-quinolone sequential therapy group, compared to 10 weeks (95% CI: 3.9–16.1 weeks) for the azithromycin group (Log-Rank test, p = 0.001) (**Figure 2**).

Notably, the mean time to clearance was substantially longer in the azithromycin group (94.2 weeks, 95% CI: 58.5–129.8 weeks) than in the doxycycline-quinolone sequential therapy group (28.0 weeks, 95% CI: 19.5–36.4 weeks), indicating a pronounced “long-tail” effect in the azithromycin cohort, where a subset of patients experienced markedly delayed clearance or persistent infection.

Consistent with the logistic regression findings, baseline co-infection with CT was strongly associated with delayed MG clearance. Patients with CT co-infection had a median time to clearance of 27 weeks (95% CI: 13.5–40.5), compared to 7 weeks (95% CI: 6.0–8.0) for those without CT (Log-rank test, p < 0.001). The mean clearance time was also substantially longer in the CT-positive group (85.4 vs. 31.5 weeks), indicating a severe “long-tail” effect in this population (**Supplementary Figure 1**).

Similarly, male sex was associated with a slower clearance trajectory. The median time to clearance was 8 weeks (95% CI: 5.9–10.1) for males compared to 6 weeks (95% CI: 4.8–7.2) for females (Log-rank test, p = 0.001). The mean clearance time was also longer in male patients (46.3 vs. 21.4 weeks) (**Supplementary Figure 2**), further supporting the increased risk of unfavorable outcomes observed in regression analyses.

### 3.6 Risk factors for recurrent infection

Finally, we assessed predictors of recurrence among the 356 patients who had achieved documented clearance of MG (**Table 5**). Male sex was again a strong and independent risk factor, associated with a nearly four-fold increase in the odds of recurrence (aOR = 3.70, 95% CI: 1.19–11.49, p=0.024). A history of baseline co-infection with CT also doubled the odds of experiencing a recurrent episode (aOR = 2.29, 95% CI: 1.09–4.82, p=0.030). Crucially, initial therapy with doxycycline-quinolone sequential therapy demonstrated a sustained protective effect, reducing the odds of recurrence by 63% compared to azithromycin (aOR = 0.37, 95% CI: 0.18–0.75, p=0.006). Age and co-infection with UU or NG were not associated with recurrence risk.

**Table 5:** Multivariable analysis of factors associated with recurrent MG infection among patients who achieved initial clearance.

Factor	Adjusted odds ratio (aOR)	95% confidence interval	P value
Age (per year increase)	1.00	0.97-1.03	0.939
Sex (Male vs. Female)	3.70	1.19-11.45	**0.024**
*U. urealyticum* co-infection (Yes vs. No)	0.64	0.30-1.37	0.245
*C. trachomatis* co-infection (Yes vs. No)	2.29	1.09-4.82	**0.030**
*N. gonorrhoeae* co-infection (Yes vs. No)	0.59	0.13-2.75	0.505
Initial antibiotic regimen (Ref: Azithromycin)			
Doxycycline-quinolone	0.37	0.18-0.75	**0.006**
Quinolone	0.51	0.13-1.93	0.318

Outcome definition (recurrent) differs from that in **Table 3**, but variable definitions, model construction, and interpretation of adjusted odds ratios otherwise follow the footnote to **Table 3**.

Bold values indicate P<0.05, considered statistically significant.

## 4 Discussion

This retrospective cohort study, based on a large clinical sample, provides a multidimensional assessment of factors influencing the treatment outcomes of MG infection. The choice of initial antibiotic regimen was decisive for long-term success. Doxycycline-quinolone sequential therapy yielded significantly higher odds of definitive cure and lower recurrence risk than azithromycin, despite comparable 8-week clearance rates. Co-infection with CT and male sex were strong, independent predictors of adverse outcomes across all timepoints. Survival analysis corroborated these findings, demonstrating markedly delayed clearance associated with azithromycin use, CT co-infection, and male sex.

The critical distinction between regimens lay in clearance dynamics. Survival analysis revealed a profound “long-tail” effect in the azithromycin group. The median time to clearance was already longer (10 vs. 7 weeks), but the disparity in mean clearance time was staggering (94.2 vs. 28.0 weeks). This pattern indicates that a substantial subset of patients receiving azithromycin experience extremely delayed or ineffective clearance, which directly translates into the higher ultimate failure rate we observed. In contrast, the doxycycline-quinolone regimen facilitated more rapid and reliable eradication. This finding aligns with and extends the rationale for “sequential therapy” advocated in settings of high macrolide resistance (Machalek et al., 2020). The observation that azithromycin achieved comparable short-term clearance but higher rates of long-term failure and recurrence suggests that its failure frequently manifests not as outright initial inefficacy, but as “protected persistence”. This phenomenon may be driven by MG’s ability to establish persistent intracellular infections. McGowin et al. demonstrated that MG can survive within human endocervical epithelial cells for extended periods, creating a protected reservoir that is likely less accessible to some antibiotics (McGowin et al., 2012). Doxycycline may serve a crucial “debulking” function, reducing bacterial load and potentially disrupting protective niches. Subsequent quinolone administration then targets the residual, potentially quiescent population. This multi-target, two-phase approach offers a biologically plausible strategy to overcome both conventional resistance and the biofilm-like or intracellular persistence states hypothesized in refractory MG infection (Yueyue et al., 2022).

Co-infection with CT emerged as the strongest independent predictor of unfavorable outcomes in MG infection (aOR 2.34–3.21). Its impact extends far beyond merely adding to the comorbidity burden; CT co-infection fundamentally alters the clearance kinetics of MG. Our survival analysis clearly highlights this prolonged infection burden: compared to CT-negative individuals, CT-positive patients experienced a nearly four-fold increase in median clearance time (27 weeks) and a 2.7-fold increase in mean clearance time (85.4 weeks). This extreme “long-tail” effect strongly suggests that CT is not a passive bystander. We propose that its prognostic value stems from a dual role: behaviorally, it acts as a robust proxy for high-risk networks with increased reinfection potential (Barnett and Brundage, 2001; Willis et al., 2021); biologically, CT-induced epithelial inflammation and altered mucosal microenvironments may facilitate MG immune evasion or persistent intracellular states (Darville and Hiltke, 2010; Yueyue et al., 2022). Thus, a positive CT status identifies a distinct clinical profile intrinsically less conducive to MG eradication.

In stark contrast to the profound impact of CT, the dynamic of UU co-detection yielded entirely different clinical implications. As articulated by Horner et al (Horner et al., 2018), distinguishing between UU infection and colonization remains challenging, with asymptomatic carriage estimated to occur in 40–80% of sexually active individuals. Consistent with this view, despite a high prevalence of UU detection in our cohort (49.1%), our multivariable analysis found no independent association between UU co-detection and MG treatment failure (**Table 3**-**5**). This null finding provides robust real-world evidence that, in the context of MG co-infection, UU represents a commensal “bystander” rather than a pathogenic modulator of clearance dynamics. These data further corroborate the position statement’s recommendation against routine testing or treatment of UU in the management of MG infections. Avoiding unnecessary UU-directed therapy mitigates the risk of antimicrobial resistance, reinforcing that clinical focus should remain firmly on established pathogens such as CT.

Male sex emerged as a robust independent predictor of poor MG outcomes, including treatment failure and recurrence (aOR range: 1.94–3.70)—an association that persisted even after adjusting for potential confounders like CT co-infection. This striking disparity likely reflects a convergence of unmeasured epidemiological dynamics and intrinsic anatomical factors. Epidemiologically, this sex disparity must be contextualized within the sexual networks of the study population. Although specific data on sexual orientation were not systematically recorded in our retrospective cohort, the “male” category inherently encompasses the subpopulation of men who have sex with men (MSM). Extensive surveillance data have consistently demonstrated that MSM harbor significantly higher rates of macrolide resistance-associated mutations and multidrug resistance compared to heterosexual men and women, driven by dense sexual networks and higher cumulative antibiotic exposure (Dionne-Odom et al., 2018; Mulligan et al., 2019). Therefore, the elevated failure rates we observed in men are likely partially attributable to a higher background prevalence of resistant strains circulating within the MSM subset of our cohort. This serves as a critical unmeasured confounder, underscoring the necessity for future prospective studies to explicitly stratify outcomes by sexual orientation. Beyond these epidemiological drivers, the higher odds of early failure (aOR=2.25) and substantially prolonged mean clearance time in males (46.3 vs. 21.4 weeks) also point to biological impediments to clearance. Anatomically, the prostate gland may act as a pharmacological sanctuary. It can sequester the organism and limit effective antibiotic penetration, allowing MG to persist despite systemic therapy (Song et al., 2024). This anatomical “protection, “ combined with the higher probability of antimicrobial resistance in the MSM population, creates a biologically plausible explanation for the treatment recalcitrance observed in male patients.

This regional resistance landscape has critical implications for interpreting our findings within the context of current clinical guidelines. Current clinical guidelines for MG management in the United States (Workowski et al., 2021), Europe (Jensen et al., 2022), the United Kingdom (Soni et al., 2019), and Australia (Ong et al., 2023) uniformly recommend resistance-guided therapy whenever feasible. Under ideal conditions, treatment selection would be strictly informed by macrolide resistance testing. However, such a strategy presupposes routine access to molecular genotyping—a precondition that remains unmet in many real-world settings, including large parts of China and other regions globally. While a global meta-analysis reported a macrolide resistance rate of 43.5% (Abavisani and Keikha, 2023), the situation in China is significantly more severe. Macrolide resistance-associated 23S rRNA mutations have reached 88.9% in Nanjing (Li et al., 2020), 66.4% in Guangzhou (Ke et al., 2020), and 83% among MSM nationwide (Wang et al., 2023). In such a high-resistance context, empirical azithromycin monotherapy is clinically untenable. While moxifloxacin remains a potent alternative, the United Kingdom guideline explicitly cautions that “Using moxifloxacin first line in all cases of *M. genitalium* is not recommended because future therapeutic options are limited” (Soni et al., 2019). This concern is particularly relevant in our region, where fluoroquinolone resistance is rising (Ke et al., 2020; Li et al., 2020).

To address this gap, we propose a risk-stratified clinical management framework grounded in two readily available clinical markers: male sex and CT co-infection. For patients identified as high-risk (men or CT-positive individuals), clinical management should be intensified through: 1) prioritizing doxycycline-quinolone sequential therapy as a first-line empirical option, particularly in regions with high macrolide resistance; 2) implementing scheduled NAAT-based TOC at defined intervals; and 3) standardizing partner notification to interrupt transmission networks. This approach is further supported by contemporary domestic guidance. The 2024 Chinese Expert Consensus on MG Infection recommends that in regions with high macrolide resistance and no access to resistance testing, treatment should follow regimens designed for macrolide-resistant infection—specifically, doxycycline followed by a fluoroquinolone (Chinese Academy of Medical Sciences Hospital of Dermatology et al., 2024). Our framework offers a critical refinement to this recommendation by incorporating risk stratification, ensuring that intensified therapy is targeted to those most likely to benefit—men and individuals with CT co-infection—thereby balancing clinical cure with the principles of antimicrobial stewardship.

Before drawing definitive conclusions, the methodological implications of our retrospective design—specifically the substantial LTFU rate of 60.2%—warrant thorough consideration regarding potential selection bias. As detailed in **Table 1**, patients in LTFU group differed from those included in the final analysis in three respects: they were slightly younger, had a higher proportion of females, and exhibited a significantly higher prevalence of UU co-infection. Several lines of evidence from our multivariable analyses suggest that these differences are unlikely to have substantively biased the primary efficacy estimates. First, while younger age showed a marginal statistical association with lower 8-week clearance (aOR 0.98 per year, p=0.033), it did not persist as an independent predictor for either ultimate treatment failure or recurrence. Similarly, despite its overrepresentation in the LTFU group, UU co-infection was not an independent predictor of treatment outcomes in any of our multivariable models. Therefore, these specific imbalances do not materially compromise our core findings. The most critical demographic difference was the underrepresentation of men in the LTFU group, resulting in a higher concentration of this high-risk demographic in our final analyzed cohort. Male sex is a strong independent risk factor for treatment failure, and their higher retention in our cohort likely inflated the observed failure rates. This effect is compounded by the ‘healthy leaver’ bias—where asymptomatically cured patients are less likely to return for a TOC—suggesting that our results represent a conservative, upper-bound estimate of the true population rates. Crucially, while this differential retention may slightly inflate the overall descriptive failure prevalence, it does not invalidate our multivariable risk factor analysis. The identification of male sex and CT co-infection as robust, independent predictors of adverse outcomes remains internally valid, as our regression models evaluate these relative associations independent of the cohort’s baseline demographic distribution.

Beyond LTFU, several other inherent limitations of this study must be acknowledged. First, the retrospective design precludes the establishment of definitive causality and is susceptible to unmeasured confounding, such as precise antibiotic adherence, detailed sexual behavior histories, and partner treatment status. Second, the lack of molecular genotyping prevented the definitive differentiation between true biological relapse and reinfection in patients with recurrent positive tests. Consequently, the reported recurrence rates represent a composite of treatment failures and new infections. However, the use of RNA-SAT confirms that these cases represented active bacterial replication rather than false-positive results from residual nucleic acids, necessitating clinical management regardless of their origin. Third, the absence of molecular resistance data (e.g., 23S rRNA and *parC* mutations) constitutes a core limitation. We cannot definitively attribute treatment failures to pre-existing macrolide or quinolone resistance, nor precisely quantify the contribution of sequential therapy in overcoming specific resistance mechanisms. This limitation underscores the current practical value of using pragmatic clinical markers—such as CT co-infection and male sex—for risk stratification, while emphasizing the critical need to integrate rapid molecular susceptibility testing in future prospective studies to achieve genuine precision therapy. Finally, the relatively small sample size in the quinolone monotherapy group and the limited number of recurrence events affect the precision of effect estimates for these specific outcomes.

Ultimately, it is crucial to acknowledge that the associations and pragmatic clinical frameworks proposed here are derived from real-world retrospective data. Their validity and broad generalizability urgently require confirmation through well-designed prospective studies. Future research should prioritize prospectively validating this risk-stratification model, integrating rapid molecular susceptibility testing to enable true precision antimicrobial stewardship, and elucidating the specific biological mechanisms underlying the profound treatment recalcitrance associated with CT co-infection and male sex.

## 5 Conclusion

This study demonstrates that, in real-world clinical practice, doxycycline-quinolone sequential therapy is associated with superior long-term cure rates and better recurrence control compared to azithromycin monotherapy for MG infection. Concurrently, it establishes co-infection with Chlamydia trachomatis and male sex as pivotal markers for clinical risk stratification. Collectively, these findings point towards an optimized management pathway: one that utilizes readily available clinical indicators (CT status, sex) for risk stratification, and prioritizes more effective initial regimens (such as sequential therapy) for high-risk patients, thereby balancing optimal clinical cure with the principles of antimicrobial stewardship. Future prospective research, integrating molecular resistance profiling and behavioral data, is essential to ultimately establish and validate this precision treatment paradigm.

## Data Availability

The original contributions presented in the study are included in the article/**Supplementary Material**. Further inquiries can be directed to the corresponding author.

## Supplementary material

Supplementary Figure 1 Kaplan–Meier curves comparing time to *Mycoplasma genitalium* clearance by baseline *Chlamydia trachomatis* (CT) co-infection status. Patients with CT co-infection (blue line) exhibited a significantly delayed clearance compared to those without CT co-infection (green line) (Log-rank test, p < 0.001). The median time to clearance was 27 weeks (95% CI: 13.5–40.5 weeks) in the CT-positive group versus 7 weeks (95% CI: 6.0–8.0 weeks) in the CT-negative group. The mean time to clearance was 85.4 weeks (95% CI: 51.3–119.5) and 31.5 weeks (95% CI: 21.6–41.5), respectively, highlighting a pronounced “long-tail” effect in the CT co-infected cohort.

Supplementary Figure 2 Kaplan–Meier curves comparing time to *Mycoplasma genitalium* clearance by sex. Male patients (blue line) demonstrated a significantly longer time to clearance compared to female patients (green line) (Log-rank test, p = 0.001). The median time to clearance was 8 weeks (95% CI: 5.9–10.1 weeks) for males versus 6 weeks (95% CI: 4.8–7.2 weeks) for females. The mean time to clearance was also prolonged in males (46.3 weeks, 95% CI: 33.1–59.5) relative to females (21.4 weeks, 95% CI: 10.6–32.1).
