# Supplementary material for: Towards precision management of Mycoplasma genitalium: a real-world cohort study identifying key predictors for treatment failure and the superiority of sequential therapy
Source: Front Cell Infect Microbiol. 2026 Apr 2;16:1787520. doi: 10.3389/fcimb.2026.1787520 (PMC13083191; doi:10.3389/fcimb.2026.1787520)
Supplement: Supplementary Figure 1 — Kaplan–Meier curves comparing time to Mycoplasma genitalium clearance by baseline Chlamydia trachomatis (CT) co-infection status. Patients with CT co-infection (blue line) exhibited a significantly delayed clearance compared to those without CT co-infection (green line) (Log-rank test, p < 0.001). The median time to clearance was 27 weeks (95% CI: 13.5–40.5 weeks) in the CT-positive group versus 7 weeks (95% CI: 6.0–8.0 weeks) in the CT-negative group. The mean time to clearance was 85.4 weeks (95% CI: 51.3–119.5) and 31.5 weeks (95% CI: 21.6–41.5), respectively, highlighting a pronounced “long-tail” effect in the CT co-infected cohort. [file Supplementaryfile1.pdf]

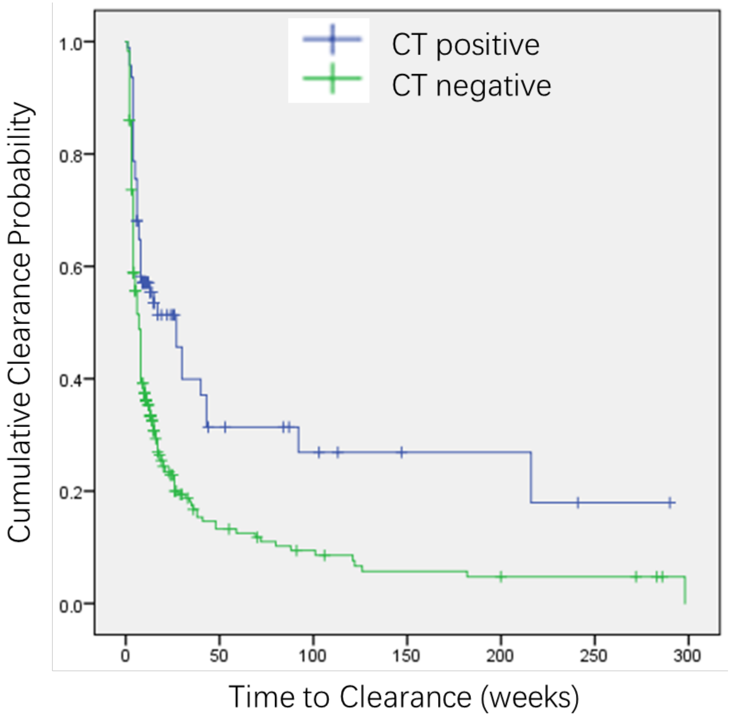

Supplementary Figure 1. Kaplan–Meier curves comparing time to *Mycoplasma genitalium* clearance by baseline *Chlamydia trachomatis* (CT) co-infection status.

Patients with CT co-infection (blue line) exhibited a significantly delayed clearance compared to those without CT co-infection (green line) (Log-rank test, $p < 0.001$). The median time to clearance was 27 weeks (95% CI: 13.5–40.5 weeks) in the CT-positive group versus 7 weeks (95% CI: 6.0–8.0 weeks) in the CT-negative group. The mean time to clearance was 85.4 weeks (95% CI: 51.3–119.5) and 31.5 weeks (95% CI: 21.6–41.5), respectively, highlighting a pronounced “long-tail” effect in the CT co-infected cohort.
