# Supplementary material for: Towards precision management of Mycoplasma genitalium: a real-world cohort study identifying key predictors for treatment failure and the superiority of sequential therapy
Source: Front Cell Infect Microbiol. 2026 Apr 2;16:1787520. doi: 10.3389/fcimb.2026.1787520 (PMC13083191; doi:10.3389/fcimb.2026.1787520)
Supplement: Supplementary Figure 2 — Kaplan–Meier curves comparing time to Mycoplasma genitalium clearance by sex. Male patients (blue line) demonstrated a significantly longer time to clearance compared to female patients (green line) (Log-rank test, p = 0.001). The median time to clearance was 8 weeks (95% CI: 5.9–10.1 weeks) for males versus 6 weeks (95% CI: 4.8–7.2 weeks) for females. The mean time to clearance was also prolonged in males (46.3 weeks, 95% CI: 33.1–59.5) relative to females (21.4 weeks, 95% CI: 10.6–32.1). [file Supplementaryfile2.pdf]

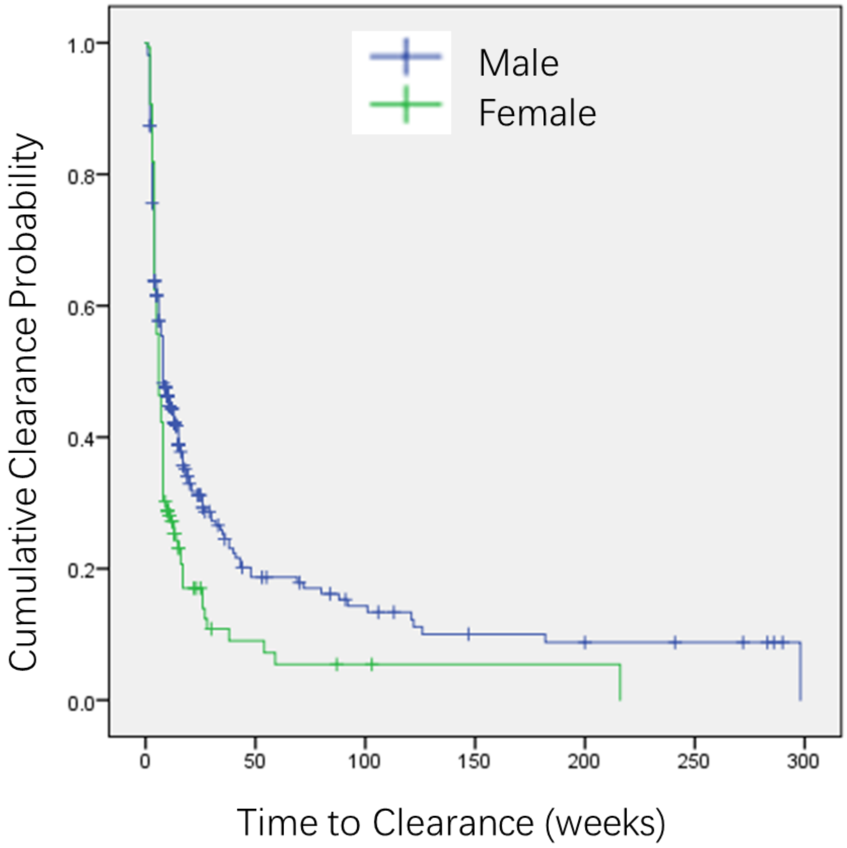

Supplementary Figure 2. Kaplan–Meier curves comparing time to *Mycoplasma genitalium* clearance by sex.

Male patients (blue line) demonstrated a significantly longer time to clearance compared to female patients (green line) (Log-rank test, $p = 0.001$). The median time to clearance was 8 weeks (95% CI: 5.9–10.1 weeks) for males versus 6 weeks (95% CI: 4.8–7.2 weeks) for females. The mean time to clearance was also prolonged in males (46.3 weeks, 95% CI: 33.1–59.5) relative to females (21.4 weeks, 95% CI: 10.6–32.1).
